# Cardiorespiratory Adaptations during Concurrent Aerobic and Strength Training in Men and Women

**DOI:** 10.1371/journal.pone.0139279

**Published:** 2015-09-29

**Authors:** Moritz Schumann, Kaisu Yli-Peltola, Chris R. Abbiss, Keijo Häkkinen

**Affiliations:** 1 Department of Biology of Physical Activity, University of Jyväskylä, Jyväskylä, Finland; 2 Centre for Exercise and Sports Science Research, School of Exercise and Health Science, Edith Cowan University, Perth, Australia; University of Rome Foro Italico, ITALY

## Abstract

This study investigated the effects of endurance followed by strength training (ES, men n = 16; women n = 15), the reverse exercise order (SE, men n = 18, women n = 13) and concurrent endurance and strength training performed on alternating days (AD, men n = 21, women n = 18) on cardiorespiratory parameters. Peak oxygen consumption ($\dot{\text{V}}$O_2peak_) and oxygen consumption at sub-maximal power outputs ($\dot{\text{V}}$O_2submax_) of 50 to 175 Watts in men and 50 to 125 Watts in women were assessed during an incremental cycling test both before and after 24 weeks of training. Increases in $\dot{\text{V}}$O_2peak_ in both men and women were statistically larger in AD (18±9% and 25±11%) compared to ES (7±9% and 12±12%, p = 0.002 and 0.009, respectively) and SE (7±9% and 10±8%, p = 0.005 and 0.008, respectively). No statistical group interaction was observed for $\dot{\text{V}}$O_2submax_ in men, but in women $\dot{\text{V}}$O_2submax_ was statistically lower at week 24 in ES compared to AD at 75 W (-2±6% vs. +3±6%, p = 0.027) and 125 W (-4±5% vs. +2±5%, p = 0.010). These findings indicate that endurance and strength training performed on alternating days may optimize the adaptations in $\dot{\text{V}}$O_2peak_ in both sexes, while performing ES training in women may optimize cardiorespiratory fitness at sub-maximal power outputs.

## Introduction

It is well established that low cardiorespiratory and neuromuscular fitness are associated with an increased mortality in both men and women [1–3]. In order to counteract a decline in physical fitness throughout the life span, global exercise recommendations suggest a combination of both endurance and strength training [4]. However, while both of these fitness aspects are important in maintaining general health many guidelines often overlook the possible complications of concurrent aerobic and resistance training, known as the interference effect [5]. Indeed, given that availability of time is the most commonly reported barrier for adherence to exercise guidelines [6], better understanding of the interference effect will aid in optimizing time-efficient exercise prescription.

Typically, the combination of endurance and strength training within the same training program results in compromised strength development and muscle hypertrophy, when the total number of weekly training sessions is high [5,7]. However, to the best of our knowledge, only one training study has yet indicated that the maximal oxygen consumption ($\dot{\text{V}}$O_2max_) may be compromised following a prolonged period (20 weeks) of concurrent endurance and strength training in men [8], while the majority of studies showed no attenuations of concurrent training-induced changes in VO_2max_ [7].

Based on previous studies it appears that adaptations to combined training may be dependent on whether endurance and strength training is performed within the same training session or on alternating days [9,10]. However, Sale et al. [10] found that improvements in $\dot{\text{V}}$O_max_ were similar when performing endurance and strength training on the same or alternating days. While these findings indicate that cardiorespiratory adaptations may be unaffected by the mode of concurrent training, $\dot{\text{V}}$O_max_ is only one aspect of cardiorespiratory function and its role as the most important predictor of cardiorespiratory fitness has previously been questioned [11]. To the best of our knowledge no prolonged training study has yet examined the influence of either combined training mode on other important cardiorespiratory adaptations such as metabolic thresholds, sub-maximal oxygen consumption or movement economy. Importantly, in the study of Sale et al. [10] no differentiation between the exercise orders (i.e. initiating a combined training session with endurance or strength) was made. Several acute studies have, however, provided evidence for reduced work economy during running for several hours following typical lower body strength loading [12–14] and during cycling following plyometric strength exercise [15]. As repeated acute reductions in exercise economy may be detrimental to beneficial cardiorespiratory adaptations in inexperienced subjects but possibly provide an additional training stimulus for athletes, the long-term effects of such training regimen remain to be investigated.

The purpose of the present study was to investigate the effects of the endurance and strength training order within the same combined training session and the effects of prolonged concurrent training performed on alternating days on cardiorespiratory fitness (i.e. $\dot{\text{V}}$O_peak_, submaximal oxygen consumption, gross efficiency and metabolic thresholds) in men and women. It was hypothesized that starting same-session combined training with resistance exercise may lead to compromised cardiorespiratory adaptations, when compared with combined endurance and strength training performed in the opposite exercise order or on alternating days. This study will expand to our previous work in which we have investigated the effects of the exercise order of same-session combined training (endurance followed by strength or vice versa) on the changes in physical fitness and body composition in physically active men [16] and women [17] and neuromuscular performance following three modes of combined training in men [10].

## Materials and Methods

### Subjects

A total of 140 healthy men (n = 70) and women (n = 70) were recruited to participate in this study. The initial health and activity status of the subjects was assessed by a standardized phone interview. Subjects were moderately active as characterized by irregular walking, cycling or occasional team sports at light to moderate intensity and not more than 3 times per week. Subjects did not engage in systematic or structured endurance or strength training prior to the study. The study was conducted according to the Declaration of Helsinki and ethical approval was granted by the Ethics Committee at the University of Jyväskylä. Subjects were informed about possible risks of all study procedures before giving written informed consent. Subjects completed a health questionnaire which along with a resting electrocardiogram was reviewed by a cardiologist prior to participating in the study. All subjects were reportedly free of acute and chronic illness, disease and injury and the use of medications that would contraindicate the performance of intense physical activity. Out of the 140 originally recruited subjects, 39 (15 men and 24 women) did not complete the study or were not included in the data analysis due to a training adherence of less than 85%. Demographic characteristics of all included subjects are presented in Table 1.

**Table 1:** Anthropometrics of subjects within ES, SE and AD.

		Age (y)	Body mass (kg)	Height (cm)	BMI
**Men**	**ES (n = 16)**	30±6	81±12	178±6	25±3
	**SE (n = 18)**	30±4	76±9	179±5	24±2
	**AD (n = 21)**	29±6	82±11	180±7	26±4
**Women**	**ES (n = 15)**	30±5	68±11	168±6	24±3
	**SE (n = 13)**	29±4	63±8	164±5	24±4
	**AD (n = 18)**	30±8	68±9	168±5	24±3

### Study design

Throughout the duration of the study subjects performed 24 weeks of supervised training. Following the health-screening, subjects were matched by physical performance to one of the following training groups: i) endurance training immediately followed by strength training (men, ES n = 16; women, ES, n = 15), ii) strength training immediately followed by endurance training (men, SE, n = 18; women, SE, n = 13), or iii) endurance and strength training performed on alternating days (men, AD, n = 21; women, AD, n = 18). As this study was aimed to investigate the differences between three different concurrent training modes, a no-training control group was not included. Prior to, as well as following 12 and 24 weeks of training the cardiorespiratory fitness and strength of subjects was assessed. Cardiorespiratory fitness and strength testing was conducted over two separate days at a similar time of day (i.e. **±**1 h). Testing at week 12 and 24 was separated from the training by at least two days of recovery. Subjects were required to maintain nutritional intake throughout the study period.

### Endurance and strength training

Subjects were asked to maintain individual habitual physical activity (e.g. light walking, cycling and occasional team sports) throughout the study period. All prescribed training in the study was supervised by qualified instructors. The training was designed to reflect a program aimed for physically active populations according to recommendations outlined by the American College of Sports Medicine [18]. The main objective was to improve physical fitness through a periodized program including both moderate and vigorous intensity aerobic exercises combined with hypertrophic and maximal strength exercise protocols.

The overall training volume was matched between all training groups so that all subjects adhered to the same training program, while the mode of endurance and strength training differed between the three groups in both men and women. Men and women assigned to ES or SE training performed 2 combined training sessions (starting with endurance or strength, respectively) per week during weeks 1–12 and 2–3 combined training sessions per week during weeks 13–24. Subjects were required to proceed from the first loading (i.e. E or S, respectively) to the subsequent loading (i.e. S or E, respectively) within 5–10 minutes. Subjects assigned to AD performed the same training volume as ES and SE but conducted endurance and strength training on alternating days (i.e. a total of 4 training sessions during weeks 1–12 and 4–6 training sessions during weeks 13–24). To avoid fatigue before testing, training load was reduced in weeks 12 and 24 by reducing training volume and intensity, i.e. decreasing the number of sets and lowering the strength training loads as well as reducing both the total duration and time spent at high intensity (i.e. above the anaerobic threshold) during endurance cycling. Training frequency was maintained during this period.

The endurance and strength training program has been described in detail elsewhere [16]. Briefly, the intensity of the endurance training was controlled by heart rate (Polar S410, Polar Electro Oy, Kempele, Finland) associated with subject’s individual aerobic and anaerobic threshold determined during the measurements at week 0 and 12, respectively. Subjects were instructed to maintain a constant pedalling frequency at approximately 70 rpm during each training session, while the magnetic resistance of the ergometer was adjusted to achieve the required exercise load. The exercise intensity progressed from steady-state cycling of low to moderate intensity (below and above the aerobic threshold) during weeks 1–7 to high-intensity interval sessions during weeks 8–12. Similarly, the duration of endurance cycling progressively increased throughout the 12 weeks of training from 30 to 50 min. During the second 12-week period the basic endurance program structure was maintained, while both training volume and intensity were further increased.

The loads used during the present strength training program were determined by the number of repetitions and execution velocity and progressively increased throughout the two 12-week periods. Exercises for the lower body were bilateral dynamic horizontal leg press as well as bilateral and unilateral dynamic knee extension and flexion. Additional exercises for the upper body included dynamic seated vertical press and lat-pull down as well as exercises commonly used to improve trunk stability (crunches, torso rotation and lower back extension). The number of sets and loads used progressively increased during the first 12 weeks of training (from 2–4 sets of 15–20 repetitions with 1 minute inter-set rest and an intensity of 40–60% of 1RM to 2–5 sets of 3–5 repetitions at 85–95% of 1 RM with 3 minutes inter-set rest), leading to a total duration of 30–50 min per training session. During the second 12-week period the basic strength program structure was maintained, while both training volume and frequency were slightly increased in order to maximize fitness and health outcomes and to avoid a training plateau.

### Cardiorespiratory fitness

After assessing body mass on a digital scale, subjects performed an incremental cycling test on a bicycle ergometer (Ergometrics 800, Ergoline, Bitz, Germany) for the determination of aerobic and anaerobic thresholds and peak ($\dot{\text{V}}$O_2peak_) and sub-maximal oxygen consumption ($\dot{\text{V}}$O_2submax_). The protocol begun at 50 W and increased by 25 W every 2 min. Subjects were asked to maintain a pedaling frequency of 70 rpm throughout the test. The test was stopped when the subjects failed to maintain the required cadence for more than 15 s. Oxygen uptake was determined breath-by-breath using a gas analyzer (Oxycon Pro, Jaeger, Hoechberg, Germany). On each testing day air flow calibration was performed using a manual flow calibrator. Before each test automatic air flow calibration was performed and the gas analyzer was calibrated using a certified gas mixture of 16% O_2_ and 4% CO_2_. $\dot{\text{V}}$O_2peak_ was calculated as the highest $\dot{\text{V}}$O_2_ value averaged over 60 s. $\dot{\text{V}}$O_2submax_ was calculated as the average $\dot{\text{V}}$O_2_ during the second minute at each power output.

Gross efficiency was calculated from the $\dot{\text{V}}$O_2_ consumption and $\dot{\text{V}}$CO_2_ production, averaged over the second minute at each power output as was previously done by Moseley and Jeukendrup [19]:

$$\text{Mechanical efficiency}=\left(\text{Power output}\;\left[\text{W}\right]\right)\;/\;\text{Energy expended}\;\left(\text{J}•{\text{s}}^{-1}\right)\;\text{x}\;100\%$$

Peak wattage was calculated as was previously done by Kuipers [20], using the equation:

$$\text{Peak wattage}={\text{W}}_{\text{com}}+\left(\text{t}/120\right)*25$$

where W_com_ is the load of the last completed stage and t is the time of the last incomplete stage in seconds.

Heart rate was monitored throughout the incremental cycling test (Polar S410, Polar Electro Oy, Kempele, Finland) with the average of the last 5 s of each stage recorded for analysis. Capillary blood samples (20 μL) were collected from the fingertip into reaction capsules containing a hemolyzing and anticoagulant agent at the end of each stage for the determination of blood lactate concentrations. Lactate concentrations were analyzed using a Biosen analyzer (C_line Clinic, EKF, Magdeburg, Germany). Individual aerobic and anaerobic thresholds for the determination of training intensities were assessed using deflection points obtained by plotting the curves of blood lactate concentrations, ventilation, oxygen consumption and carbon dioxide production [21]. For objective statistical comparisons of the three training modes, the power output and heart rate at 4 mmol L^-1^ blood lactate concentrations (OBLA) were also determined [22].

### Allometric scaling

Typically peak and sub-maximal oxygen consumption have been expressed as milliliters per kilogram (of body mass) per minute. However, previous research has shown that the relationship between oxygen uptake and body mass is not linear [23,24]. As such, allometrically scaling oxygen consumption may provide a better indication of training induced changes. Although considerable training-induced changes in body mass were not expected in the present study, peak and sub-maximal oxygen consumption was reported and analyzed both as absolute values and scaled to body mass to the power of 0.75 (ml kg^-75^ min^-1^) [24].

### Strength testing

One repetition maximum (1RM) of leg extensors was determined using a dynamic horizontal bilateral leg press device (David 210, David Health Solutions, Helsinki, Finland). Following a warm up (1 set of 5 repetitions at 70% of estimated 1RM, 1 set of 2 repetitions at 80–85% of estimated 1RM, 1 set of 1 repetition at 90–95% of estimated 1RM) a maximum of 5 trials was allowed to obtain a true 1RM. The device was set up so that the knee angle in the initial flexed position was approximately 60 degrees and a successful trial was accepted when the knees were fully extended (~180 degrees). The greatest load that the subject could lift to full knee extension at an accuracy of 1.25 kg was accepted as 1RM.

### Statistical Analyses

Data are presented as mean±SD. After assessing the normality of distribution, within and between-group differences of all variables were examined using a mixed ANOVA with repeated measures and the post-hoc analyses were performed using Bonferroni adjustments. In order adjust the data for baseline values, the absolute values observed at week 0 were subtracted from the corresponding values of week 12 and 24. Oxygen consumption, gross efficiency, heart rate and blood lactate concentrations of sub-maximal workloads during the incremental bike ergometer test were analyzed from loads completed by all subjects (i.e. 50, 75, 100, 125, 150 and 175 W in men and 50, 75, 100 and 125 W in women). Statistical significance for all tests was set at p≤0.05, while values ≤0.07 were accepted as a trend. Data were analyzed using IBM SPSS Predictive Analytics (version 20.0, IBM Inc., Chicago, IL, USA).

## Results

### Training adherence

The mean training adherence in men was 99**±**2%, 99**±**2% and 100**±**1% in ES, SE and AD, respectively. The mean training adherence in women was 98**±**4%, 99**±**2% and 99**±**2% in ES, SE and AD, respectively.

### Body mass

Body mass at baseline is presented in Table 1. Body mass remained statistically unaltered in all training groups. In women, a trend for a difference between the changes in AD compared to SE (-2**±**5 vs. +2**±**4%, p = 0.067) was observed at week 24.

### Cardiorespiratory fitness

Peak oxygen consumption ($\dot{\text{V}}$O_2peak_) and peak wattage is presented in Table 2. $\dot{\text{V}}$O_2peak_ scaled to body mass to the power of 0.75 increased in all groups after training. Compared to baseline, improvements in scaled $\dot{\text{V}}$O_2peak_ in both men and women at week 24 were statistically larger in AD (18**±**9% and 25**±**11%) compared to ES (7**±**9% and 12**±**12%, p = 0.002 and 0.009, respectively) and SE (7**±**9% and 10**±**8%, p = 0.005 and 0.008, respectively). No statistical between-group differences were observed between ES and SE in men and women.

**Table 2:** Peak oxygen consumption, peak wattage and 1RM strength in dynamic leg press prior to (0), at 12 weeks and at 24 weeks following ES, SE and AD.

		Week 0	Week 12	Week 24
**Men**	**ES**			
	$\dot{\text{V}}$O_2peak_ (L min^-1^)	3.38±0.50	3.54±0.48* †	3.59±0.55* †
	$\dot{\text{V}}$O_2peak_ (ml kg^-1^ min^-1^)	42.2±7.2	44.0±6.3#	44.6±5.1*
	$\dot{\text{V}}$O_2peak_ (ml kg^-75^ min^-1^)	126.07±19.5†	131.6±17.3* †	133.3±15.2* †
	Peak wattage (W)	268±39	287±38*	300±38* §
	1 RM (kg)	157±30	170±27*	175±27* §
	**SE**			
	$\dot{\text{V}}$O_2peak_ (L min^-1^)	3.20±0.44	3.44±0.41*	3.42±0.35* †
	$\dot{\text{V}}$O_2peak_ (ml kg^-1^ min^-1^)	42.5±7.0	45.2±6.9*	45.3±6.9*
	$\dot{\text{V}}$O_2peak_ (ml kg^-75^ min^-1^)	125.0±19.0†	133.4±18.2*	133.3±17.9* †
	Peak wattage (W)	245±35	268±37*	284±37* §
	1 RM (kg)	143±23	160±21*	166±20* §
	**AD**			
	$\dot{\text{V}}$O_2peak_ (L min^-1^)	2.92±0.36	3.30±0.34*	3.39±0.34*
	$\dot{\text{V}}$O_2peak_ (ml kg^-1^ min^-1^)	36.2±6.5	40.1±6.3*	42.4±6.6* §
	$\dot{\text{V}}$O_2peak_ (ml kg^-75^ min^-1^)	108.4±17.0	122.1±17.3*	126.5±16.8*
	Peak wattage (W)	233±30	264±25*	279±28* §
	1 RM (kg)	142±24	156±21*	159±22* §
**Women**	**ES**			
	$\dot{\text{V}}$O_2peak_ (L min^-1^)	2.07±0.36	2.23±0.34*	2.31±0.27* †
	$\dot{\text{V}}$O_2peak_ (ml kg^-1^ min^-1^)	30.7±3.8	33.0±4.3*	34.0±4.0*
	$\dot{\text{V}}$O_2peak_ (ml kg^-75^ min^-1^)	87.7±10.7	94.0±11.1*	97.5±9.7* †
	Peak wattage (W)	170±26	189±25*	204±23* §
	1 RM (kg)	102±22	109±23* †	115±23* §
	**SE**			
	$\dot{\text{V}}$O_2peak_ (L min^-1^)	2.12±0.32	2.29±0.29*	2.35±0.28* †
	$\dot{\text{V}}$O_2peak_ (ml kg^-1^ min^-1^)	33.8±4.7	36.1±5.3	36.9±4.8*
	$\dot{\text{V}}$O_2peak_ (ml kg^-75^ min^-1^)	94.9±12.4†	101.7±12.9*	104.1±11.7* †
	Peak wattage (W)	182±27	192±25	210±26* §
	1 RM (kg)	100±18	109±18*	116±17* §
	**AD**			
	$\dot{\text{V}}$O_2peak_ (L min^-1^)	1.86±0.25	2.14±0.22*	2.26±0.23*
	$\dot{\text{V}}$O_2peak_ (ml kg^-1^ min^-1^)	27.9±5.8	32.1±4.8*	34.7±5.8* §
	$\dot{\text{V}}$O_2peak_ (ml kg^-75^ min^-1^)	79.6±13.5†	91.6±11.9*	98.5±13.8* §
	Peak wattage (W)	155±25	179±20*	188±29*
	1 RM (kg)	88±12	103±14*	106±14* §

*≤0.05 compared to corresponding value of week 0

§ p≤0.05 compared to corresponding value of week 12

† p≤0.05 value at week 0 and change at week 12 or 24 compared to AD

# statistical trend p≤0.06

**Fig 1:**
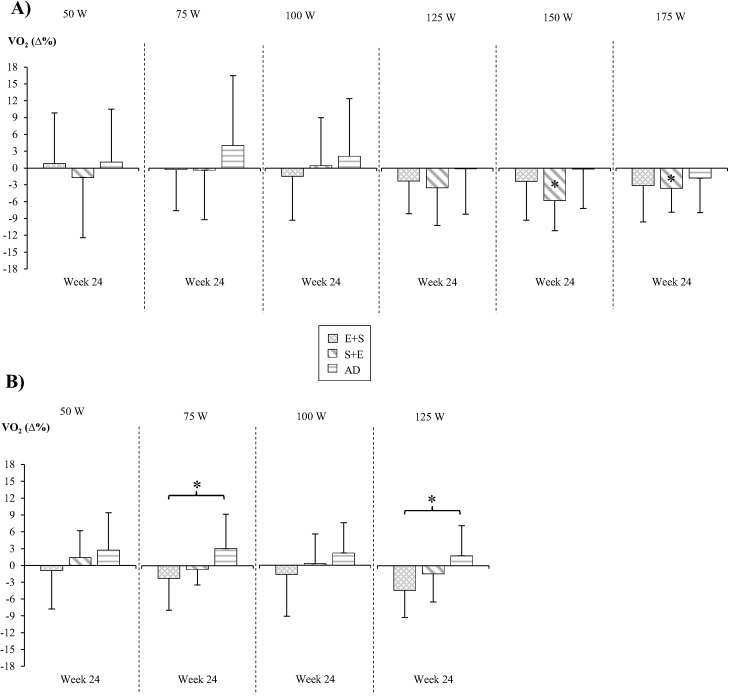
Oxygen consumption at sub-maximal power outputs in men (A) and women (B). * p<0.05; within the bar compared to corresponding value at week 0, outside the bar as indicated.

Absolute values of sub-maximal oxygen consumption are presented in Table 3. Statistical reductions in sub-maximal oxygen consumption scaled to body mass to the power of 0.75 (Fig 1) at week 24 were only found in men at 150 and 175 W (-6±5%, p = 0.002 and -4±4%, p = 0.010, respectively) but not women. No statistical group interaction was observed for sub-maximal oxygen consumption in men. However, in women statistical between-group differences at week 24 were observed between ES and AD (75W -2±6% vs. +3±6%, p = 0.027; 125 W -4±5% vs. +2±5%, p = 0.010).

**Table 3:** Sub-maximal oxygen consumption ($\dot{\text{V}}$O_2_) during the incremental cycling test performed prior to (0), at 12 weeks and at 24 weeks following ES, SE and AD.

		$\dot{\text{V}}$O_2_ (L min^-1^)					
		50W	75W	100W	125W	150W	175W
**Men**	**ES**						
	0	1.05±0.07	1.29±0.07	1.52±0.08	1.77±0.07	2.01±0.08	2.31±0.09
	12	1.06±0.10	1.27±0.10† •	1.49±0.09	1.72±0.09	1.99±0.10	2.26±0.10
	24	1.05±0.12	1.28±0.10	1.49±0.09	1.72±0.11	1.95±0.13	2.23±0.12
	**SE**						
	0	1.03±0.10	1.25±0.08	1.47±0.08	1.76±0.08	2.06±0.08	2.33±0.09
	12	1.08±0.08	1.30±0.07	1.52±0.07	1.76±0.06	2.02±0.06	2.32±0.07
	24	1.01±0.07	1.25±0.08	1.48±0.09	1.70±0.09	1.95±0.09*	2.26±0.07#
	**AD**						
	0	1.01±0.06	1.20±0.09	1.45±0.09	1.71±0.10	1.97±0.12	2.25±0.12
	12	1.04±0.09	1.25±0.07	1.46±0.05	1.71±0.07	1.96±0.06	2.21±0.06
	24	1.00±0.06	1.23±0.07	1.46±0.08	1.68±0.09	1.94±0.06	2.18±0.07*
**Women**	**ES**						
	0	0.93±0.08	1.13±0.08	1.36±0.05	1.63±0.07		
	12	0.91±0.08	1.13±0.10	1.34±0.08	1.58±0.12•		
	24	0.92±0.09	1.12±0.10	1.35±0.09	1.57±0.10		
	**SE**						
	0	0.91±0.07	1.13±0.08	1.36±0.09	1.61±0.10		
	12	0.96±0.08	1.17±0.09	1.41±0.10†	1.65±0.11†		
	24	0.93±0.07	1.13±0.09	1.37±0.08	1.60±0.11		
	**AD**						
	0	0.91±0.06	1.12±0.06	1.35±0.06	1.60±0.06		
	12	0.92±0.06	1.11±0.05	1.32±0.05	1.56±0.06		
	24	0.92±0.07	1.13±0.05	1.35±0.05	1.59±0.06		

* p≤0.05 compared to corresponding values of week 0

# statistical trend p≤0.07 compared to corresponding values of week 0

† p≤0.05 change from week 0 compared to AD

• p≤0.05 change from week 0 compared to SE

Gross efficiency is presented in Table 4. Increases at week 24 were observed in men in ES at 125 W, 150 W and 175W (4±6% to 5+6%, p = 0.069 to 0.017), in SE at 150 and 175W (3±3 to 4±6%, p = 0.019 to 0.003) and in AD at 175 W (4±6%, p = 0.020) only. In women, a statistical increase was observed at week 24 in ES at 125 W only (5±6%, p = 0.017). No statistical group interaction was observed for gross efficiency in men. Statistical between-group differences were only observed in women at week 12 between ES and SE (50 W, 75 W and 125 W, +2±5% to +3±6% vs. -3±7% to -5±8%, p = 0.011 to 0.050) and between SE and AD (75 W, 100 W and 125 W, -2±7% to -3±7% vs. +2±5% to 3±4%, p = 0.033 to 0.054).

**Table 4:** Gross efficiency during the incremental cycling test performed prior to (0), at 12 weeks and at 24 weeks following ES, SE and AD.

		Gross efficiency (%)					
		50W	75W	100W	125W	150W	175W
**Men**	**ES**						
	0	14.1±1.0	16.9±1.0	18.9±0.9	20.2±0.7	21.1±0.9	21.3±0.9
	12	14.0±1.2	17.3±1.4	19.4±1.1	20.9±1.1*	21.6±1.0	22.0±0.9
	24	14.1±1.5	17.2±1.2	19.4±1.1	20.9±1.3#	21.9±1.4#	22.2±1.1*
	**SE**						
	0	14.2±1.2	17.3±1.0	19.4±0.9	20.1±0.9	20.5±0.8	21.0±0.9
	12	13.6±1.1	16.8±0.9	19.0±0.8	20.3±0.6	21.0±0.6	21.2±0.5
	24	14.5±1.1	17.5±1.1	19.4±1.2	20.9±1.2	21.8±1.1*	21.8±0.6*
	**AD**						
	0	14.5±0.9	18.1±1.4	19.7±1.3	20.6±1.3	21.3±1.5	21.6±1.4
	12	14.1±1.3	17.3±1.0	19.6±0.8	20.7±1.0	21.6±0.7	22.2±0.7
	24	14.6±1.0	17.8±1.2	19.7±1.2	21.2±1.3	21.9±0.7	22.5±0.8*
**Women**	**ES**						
	0	15.9±1.3	19.0±1.2	20.7±0.7	21.3±0.8		
	12	16.3±1.5•	19.3±1.6•	21.2±1.1	22.2±1.3* •		
	24	16.0±1.9	19.4±1.8	21.1±1.5	22.3±1.4*		
	**SE**						
	0	16.2±1.4	19.3±1.4	21.0±1.5	21.7±1.5		
	12	15.4±1.3	18.6±1.4†	20.3±1.2†	21.3±1.2†		
	24	15.9±1.3	19.3±1.5	20.9±1.2	22.1±1.4		
	**AD**						
	0	16.1±1.0	19.2±1.0	20.8±0.9	21.6±0.8		
	12	16.0±1.0	19.5±0.9	21.3±0.9	22.3±0.9*		
	24	16.0±1.1	19.1±0.8	21.0±0.7	22.0±0.8		

* p≤0.05 compared to corresponding values of week 0

# statistical trend p≤0.07 compared to corresponding values of week 0

† p≤0.05 change from week 0 compared to AD

• p≤0.05 change from week 0 compared to SE

Sub-maximal heart rate across all sub-maximal power outputs statistically decreased in men after training in ES (-5±13% to -8±5%, p = 0.018 to p<0.001), SE (-6±12% to -8±7%, p = 0.042 to p = 0.001) and AD (-8±8% to 11±7%, p<0.001). In women, heart rate statistically decreased after training only in ES (-5±8% to -8±10%, p = 0.066 to 0.001) and AD (-6±10% to -8±8% p = 0.063 to 0.001). However, no statistical group interactions were observed in men or women.

Sub-maximal blood lactate concentrations were lower for both men and women in all three training groups (Table 5) after training. No group interactions were observed in men or women.

**Table 5:** Blood lactate concentrations at sub-maximal intensities during the incremental cycling test performed prior to (0), at 12 weeks and at 24 weeks following ES, SE and AD.

		Blood lactate (mmol L^-1^)					
		50W	75W	100W	125W	150W	175W
**Men**	**ES**						
	0	1.3±0.5	1.3±0.5	1.6±0.5	1.9±0.8	2.7±1.1	3.4±1.5
	12	1.2±0.3•	1.3±0.3	1.4±0.4	1.8±0.5	2.3±0.7	2.9±1.1
	24	1.2±0.5	1.1±0.5	1.3±0.5#	1.6±0.7	2.0±1.0*	2.8±1.4†
	**SE**						
	0	1.8±0.5	1.8±0.5	2.2±0.5	3.0±1.0	4.1±2.0	4.7±1.7
	12	1.3±0.4*	1.5±0.4	1.8±0.4#	2.3±0.7	3.1±1.1	4.0±1.7
	24	1.3±0.4*	1.4±0.4*	1.6±0.3*	2.0±0.4*	2.6±0.7*	3.3±1.1*
	**AD**						
	0	1.1±0.3	1.5±0.5	2.1±0.9	2.9±1.4	3.7±1.0	5.1±1.3
	12	1.2±0.3	1.3±0.4*	1.7±0.6*	2.2±0.8*	2.8±0.7*	3.8±0.9*
	24	1.3±0.5	1.4±0.5	1.7±0.7*	2.1±0.9*	2.6±0.8*	3.4±1.0*
**Women**	**ES**						
	0	1.5±0.4	2.1±0.8	3.1±1.2	4.6±1.8		
	12	1.3±0.2	1.7±0.4	2.3±0.7	3.5±1.3		
	24	1.3±0.2	1.7±0.4	2.3±0.7*	3.5±1.3*		
	**SE**						
	0	1.3±0.4	1.8±0.5	2.6±1.0	4.0±1.7		
	12	1.4±0.5† †	1.8±0.9	2.3±1.1	3.5±1.7*		
	24	1.1±0.3	1.4±0.5*	1.9±0.9*	3.0±1.4*		
	**AD**						
	0	1.1±0.4	1.9±0.8	3.1±1.1	4.7±1.5		
	12	1.1±0.3	1.5±0.4#	2.4±0.8*	3.5±1.0*		
	24	1.4±0.5	1.7±0.5	2.4±0.7*	3.4±1.1*		

* p≤0.05 compared to corresponding values of week 0

# statistical trend p≤0.06 compared to corresponding values of week 0

† p≤0.05 change from week 0 compared to AD

• p≤0.05 change from week 0 compared to SE

### Heart rate and power output at OBLA

Heart rate did not statistically change at OBLA in either of the training groups in men or women. No significant changes in heart rate at blood lactate concentration of 4 mmol L^-1^ were observed in any training group. In men the power output at OBLA statistically increased after training in SE (25±21%, p<0.001) and AD (21±15%, p<0.001) but not ES (10±15%, p>0.05). Similarly, in women power output at OBLA increased after training in SE (13±11%, p = 0.001) and AD (18±16%, p<0.001) but not ES (15±19%, p>0.05). However, no statistical group interaction was observed.

### Strength performance

Dynamic leg press 1RM is presented in Table 2. Maximal strength improved in all groups after training. In men, no statistical group interaction was observed. However, in women a greater increase in 1RM strength was observed at week 12 in AD compared to ES (17±7% vs. 7±10%, p = 0.015).

## Discussion

The main findings of this study were that: i) $\dot{\text{V}}$O_2peak_ increased to a statistically larger extent when endurance and strength training were performed on alternating days, compared with endurance and strength training combined within the same training session (SE and ES) in both men and women and ii) adaptations in sub-maximal oxygen consumption did not statistically differ between the three training modes in men, while in women $\dot{\text{V}}$O_submax_ decreased to a larger extent in ES compared to AD.

The observed increases in $\dot{\text{V}}$O_2peak_ in AD (18% and 25%) were twice as large as those observed in ES (7% and 12%) and SE (7% and 10%) in both men and women. These findings differ from a previous study of Sale et al. [10], who did not observe differences in cardiorespiratory adaptations between combined training performed within the same session or on alternating days. The difference between these two studies, however, may be attributed to the specific training performed. Even though the endurance intensity and duration of the present training was moderate during the first half of each training period, the loads used during the strength training were heavy to maximal throughout most of the training weeks. In contrast, strength training in the study of Sale et al. [10] was performed using low loads and a large number of 15–20 repetitions, resulting in an endurance style loading. In fact, our finding of larger increases in $\dot{\text{V}}$O_2peak_ in AD compared to ES and SE are in line with previous data of Robineau et al. [25] and possibly indicate that the presence of heavy strength training performed in close proximity to the endurance loading of same-session combined training might lead to compromised adaptations in maximal oxygen consumption. However, the exact mechanisms for this finding cannot be elucidated with the present study design. It is possible that the observed attenuated changes in $\dot{\text{V}}$O_2peak_ in ES and SE may have been attributed to a state of overreaching/overtraining, induced by the relatively high volume of each combined training session (i.e. a total duration of 60–100 min per training session) for previously untrained subjects. On the other hand, the larger increases in $\dot{\text{V}}$O_2peak_ in the AD group may also be attributed to a larger weekly training frequency (i.e. 4–6 sessions compared to 2–3 sessions in ES and SE) and, thus, more frequent training stimuli, despite the overall training volume being matched. Irrespective of the cause for the present findings, however, it is important to note that $\dot{\text{V}}$O_2peak_ levels at baseline in both men and women were statistically lower in AD compared to ES and SE when normalized to body mass. Even though subjects were originally matched by physical performance, statistical between-group differences at baseline occurred due to drop outs. While our statistical approach accounted for this initial difference, the physiological window of adaptations was certainly larger in the AD groups and, thus a larger magnitude of adaptations may be expected. Yet, we are confident that this cannot fully account for the observed large between-group differences after 24 weeks of training.

Interestingly, even though $\dot{\text{V}}$O_2peak_ improved to a larger extent in AD, in women the largest decreases in oxygen consumption at sub-maximal loads occurred in the ES training group (Table 3). Moreover, while the reductions in $\dot{\text{V}}$O_2submax_ were somewhat lower in ES compared with SE, a statistical between-group difference was observed between ES and AD only. While it would typically be expected that prolonged endurance training leads to increases in maximal oxygen consumption and exercise economy, previously an inverse relationship has been observed in elite cyclists [26]. Among several hypotheses, it has been suggested that subjects with a higher $\dot{\text{V}}$O_2max_ would also tend to have higher lipid oxidation at any sub-maximal work level. Thus, as lipid oxidation releases less energy per liter of oxygen consumed, more oxygen would be required to sustain a given power output and, thus, increase exercise economy [27]. In addition, Sale et al. [10] have shown that citrate synthesis was increased when endurance and strength training where combined within the same session compared to alternating days. While it is reasonable to assume that enhanced citrate synthesis may lead to increased exercise economy, Hunter et al. [28] have shown an inverse relationship between the oxidative capacity and walking economy in premenopausal women. Thus, the mechanisms responsible for the observed difference in sub-maximal oxygen consumption between ES and AD training in women in the present study require further investigation. Moreover, when interpreting the findings of the present study, it should be acknowledged that the measurement duration for sub-maximal oxygen consumption at each power output was relatively short, which may in turn have an effect on the obtained $\dot{\text{V}}$O values. However, even relatively short increments have previously been shown to provide valid data of sub-maximal cardiorespiratory parameters [19,29]. While being aware that typically longer durations of a constant load are desired for the determination of sub-maximal physiological data (i.e. exercise economy) and shorter durations for the assessment of VO_2peak_ (i.e. a ramp test), the present incremental test was chosen in an attempt to test both maximal and sub-maximal performance indicators within the same protocol.

In contrast to our hypothesis, no statistical difference in the changes of peak and sub-maximal oxygen consumption between the ES and SE in men and women after the training was observed. This finding was somewhat surprising as previous cross-sectional studies have shown that movement economy may be reduced for several hours following strength exercise [12,14,15]. Possibly as a result, Chtara et al. [30] showed that in young men endurance performance and cardiorespiratory fitness increased to a larger extent following ES compared to SE training. However, Drummond et al. [31] suggested that endurance running performed immediately after a strength training session may actually act as an active recovery strategy following the strength loading by enhancing lactate removal [32]. Thus, performing strength exercise prior to endurance loading may not ultimately lead to compromised short or long-term cardiorespiratory function. Moreover, it should be acknowledged that even a large magnitude of exercise-induced metabolic stress (e.g. reflected in larger EPOC responses) may induce increases in cardiorespiratory fitness (i.e. maximal and sub-maximal oxygen consumption), if sufficient recovery is provided. As the training frequency of 2–3 weekly combined sessions in the SE and ES groups was relatively low, our training program allowed for at least 48h of recovery between subsequent sessions, and thus may have been sufficient for positive adaptations to take place irrespective of the exercise order. Moreover, it should be noted that the training program of the present study utilized a progressive increase in endurance and strength training volume and intensity. While all training groups improved aerobic capacity and muscle strength to an expected degree, it is possible that the weekly training stimulus may have not been sufficient for group-specific differences to occur. In addition, it should be noted that the present strength training protocol for the lower body consisted of dynamic leg press, knee extension and knee flexion protocols, all of which are similar to prolonged endurance cycling in terms of the muscles loaded and time under tension [33] and, thus, endurance and strength training in the same-session combined groups may have led to cumulative rather than adverse acute effects.

Interestingly, even though a statistical between-group difference in $\dot{\text{V}}$O_2submax_ in women was observed, no systematic statistic reductions in sub-maximal oxygen consumption were observed in either of the training groups in men and women but gross efficiency statistically increased in all training groups in men. Typically the metabolic cost of cycling at sub-maximal loads decreases when aerobic capacity is increased by prolonged training. In addition, strength training has previously been shown to induce positive adaptations in cycling economy in previously untrained men[34]. These strength training-induced adaptations have been attributed to metabolic adaptations such as increased phosphocreatine, lower lactate and higher glycogen content during exercise[35], increased peak muscle tension[36], increased tendon cross-sectional area [37] or delayed muscle fatigue[38]. Furthermore, Cadore et al. [39]have shown that strength training may improve neuromuscular economy as reflected by decreased EMG at different power outputs during cycle ergometry in elderly subjects. While such measurement was beyond the scope of the present study, all training groups statistically improved 1RM strength performance after 24 weeks of training but this was not reflected in $\dot{\text{V}}$O_2submax_ in either of the training groups and gross efficiency in women. However, blood lactate concentrations statistically decreased at most power outputs in both men and women, indicating a positive training adaptation.

Interestingly, in contrast to the beneficial adaptations in sub-maximal oxygen consumption in ES in women, this finding was not observed in men. Even though not statically different, in men the magnitude of improvement of the load at OBLA in ES was only half as large (10±15%) as that observed in SE (25±21%) and AD (21±15%). Previous studies have shown that the oxygen demands following combined training sessions are increased when endurance exercise preceded strength loadings [31,40]. However, typically this has been considered as a positive exercise response by supporting adenosine triphosphate and creatine phosphate resynthesis, the replenishment of glycogen and oxygen stores and lactate removal[41]. Our findings of compromised development of the load at the metabolic threshold following ES training in men emphasizes the need for further investigation as to what may be the possible causes.

## Perspective

This study showed that the training mode of combined endurance and strength training affected $\dot{\text{V}}$O_2peak_ in men and both $\dot{\text{V}}$O_2peak_ and $\dot{\text{V}}$O_2submax_ in women. Our findings indicate that endurance and strength training performed on alternating days may optimize the adaptations in $\dot{\text{V}}$O_2peak_ in both sexes, possibly attributed to prolonged recovery (i.e. at least 24 h) between subsequent training sessions. However, in women largest reductions in $\dot{\text{V}}$O_2submax_ were observed following ES training. Therefore, as most activities of daily living only require a fraction of the peak oxygen consumption, in women the ES exercise sequence may be desired over combined training performed with the opposite exercise order or on alternating days in order to optimize cardiorespiratory fitness.
